# Anti-cancer effect of afatinib, dual inhibitor of HER2 and EGFR, on novel mutation *HER2* E401G in models of patient-derived cancer

**DOI:** 10.1186/s12885-022-10428-3

**Published:** 2023-01-23

**Authors:** Yohei Harada, Akemi Sato, Hideaki Nakamura, Keita Kai, Sho Kitamura, Tomomi Nakamura, Yuki Kurihara, Sadakatsu Ikeda, Eisaburo Sueoka, Shinya Kimura, Naoko Sueoka-Aragane

**Affiliations:** 1 Division of Hematology, Respiratory Medicine and Oncology, Department of Internal Medicine, Faculty of Medicine, Saga University, 5-1-1 Nabeshima, Saga, 849-8501 Japan; 2 Graduate School of Medicine, Kyoto University, 53 Shogoin-Kawaharacho, Sakyo-ku, Kyoto, 606-8507 Japan; 3 Department of Clinical Laboratory Medicine, Faculty of Medicine, Saga University, 5-1-1 Nabeshima, Saga, 849-8501 Japan; 4 Department of Transfusion Medicine, Saga University Hospital, 5-1-1 Nabeshima, Saga, 849-8501 Japan; 5 Department of Pathology, Saga University Hospital, 5-1-1 Nabeshima, Saga, 849-8501 Japan; 6 Department of Precision Cancer Medicine, Center for Innovative Cancer Treatment, Tokyo Medical and Dental University, 1-5-45 Yushima, Bunkyo-ku, Tokyo, 113-8510 Japan

**Keywords:** HER2, Extracellular domain, EGFR, Afatinib, Patient-derived cancer model

## Abstract

**Background:**

Precision medicine with gene panel testing based on next-generation sequencing for patients with cancer is being used increasingly in clinical practice. *HER2*, which encodes the human epidermal growth factor receptor 2 (HER2), is a potentially important driver gene. However, therapeutic strategies aimed at mutations in the *HER2* extracellular domain have not been clarified. We therefore investigated the effect of EGFR co-targeted therapy with HER2 on patient-derived cancer models with the *HER2* extracellular domain mutation E401G, based on our previous findings that this mutation has an epidermal growth factor receptor (EGFR)-mediated activation mechanism.

**Methods:**

We generated a xenograft (PDX) and a cancer tissue-originated spheroid (CTOS) from a patient’s cancer containing an amplified *HER2* E401G mutation. With these platforms, we compared the efficacy of afatinib, a tyrosine kinase inhibitor having anti-HER2 and anti-EGFR activity, with two other therapeutic options: lapatinib, which has similar properties but weaker EGFR inhibition, and trastuzumab plus pertuzumab, for which evidence exists of treatment efficacy against cancers with wild-type *HER2* amplification. Similar experiments were also performed with H2170, a cell line with wild-type *HER2* amplification, to contrast the characteristics of these drug’s efficacies against *HER2* E401G.

**Results:**

We confirmed that PDX and CTOS retained morphological and immunohistochemical characteristics and *HER2* gene profiles of the original tumor. In both PDX and CTOS, afatinib reduced tumor size more than lapatinib or trastuzumab plus pertuzumab. In addition, afatinib treatment resulted in a statistically significant reduction in *HER2* copy number at the end of treatment. On the other hand, in H2170 xenografts with wild-type *HER2* amplification, trastuzumab plus pertuzumab was most effective.

**Conclusions:**

Afatinib, a dual inhibitor of HER2 and EGFR, showed a promising effect on cancers with amplified *HER2* E401G, which have an EGFR-mediated activation mechanism. Analysis of the activation mechanisms of mutations and development of therapeutic strategies based on those mechanisms are critical in precision medicine for cancer patients.

## Background

Multi-gene panel testing of cancer by using next-generation sequencing (NGS) is now widespread, leading to proposals for optimal treatment of individual cancer patients in the clinical practice of oncology [1, 2]. Therapeutic approaches targeting driver gene abnormalities have been separately developed for each cancer type [3], but the basket trials, which examine whether a molecular targeted agent works for various types of cancers with the same driver gene abnormalities, are increasing [4]. Variants of the *HER2* gene, which encodes the human epidermal growth factor receptor 2 (HER2) protein, are one of the most promising therapeutic targets. The MyPathway trial showed that patients with *HER2* amplification benefited from a combination of trastuzumab and pertuzumab even if they had cancers other than those of the stomach and breast in which *HER2*-targeted therapies have been conventionally approved [5]. *HER2* mutations, especially those in the intracellular kinase domain, have been considered to be an appropriate molecular target [6–8]. The SUMMIT trial, a basket trial of neratinib targeting *HER2* and *HER3* mutations, showed some efficacy. *HER2* mutations are most frequently observed in the kinase domain; however, the potential therapeutic significance of *HER2* extracellular domain mutations other than S310F, a hot spot mutation in the extracellular domain, have not been clarified [9].

We recently experienced a case of carcinoma of unknown primary with *HER2* E401G, a variant of unknown significance (VUS) in the extracellular domain concomitant with *HER2* amplification. Comprehensive analysis using cell line and animal models, as well as molecular dynamic simulation in silico, showed that *HER2* E401G has an epidermal growth factor receptor (EGFR)-mediated activation mechanism, the same as *HER2* S310F [10]. Determining therapeutic strategies in accordance with the activation mechanism of variants is an important challenge for precision medicine based on the cancer genome, which we aimed to put into practice in this case. We hypothesized that a treatment strategy that suppresses EGFR signaling simultaneously with HER2 might be promising for *HER2* E401G based on the activation mechanism. To assess this hypothesis, we examined the efficacy of afatinib, an irreversible multitargeted tyrosine kinase inhibitor (TKI) of EGFR, HER2, and HER4 [11, 12], and we compared it to other drugs: lapatinib, which is also a TKI of EGFR and HER2 but weaker than afatinib in its EGFR inhibitory effect [12], and trastuzumab and pertuzumab, which have been demonstrated to be effective for treating cancers with wild-type *HER2* amplification.

Evaluation of drug efficacy was performed primarily with cancer models based on patient-derived specimens, the patient-derived xenograft (PDX) and the cancer tissue-originated spheroid (CTOS).

## Methods

### Materials

Human lung cancer cell line NCI-H2170 was purchased from the American Type Culture Collection (ATCC, Manassas, VA, USA); cells were cultured in RPMI-1640 medium supplemented with 10% FBS. Afatinib and lapatinib were purchased from LC laboratories (Woburn, MA, USA), and trastuzumab and pertuzumab were purchased from Chugai Pharmaceutical Co. (Tokyo, Japan). Immunodeficient Balb/c Rag-2^−/−^ Jak3^−/−^ (BRJ) mice, which lack mature T and B lymphocytes and natural killer cells, were provided by Prof. Seiji Okada (Kumamoto University, Kumamoto, Japan).

### Generation of patient-derived xenograft and cancer-tissue originated spheroid

To examine drug efficacy of the agents mentioned above, we established PDX using cancer tissue obtained from the patient of unknown primary. The cancer tissue used to prepare the cancer models was obtained from a portion of a skin biopsy specimen collected for clinical use with Institutional Review Board approval (2020–07-R-10) and written informed consent from the patient for use for this research. The cancer tissue was subcutaneously transplanted into the dorsal flanks of 7-week-old female BRJ mice (*n* = 2) under anesthesia with midazolam, medetomidine, and butorphanol tartrate, and three or four in vivo passages were performed to establish PDX for drug efficacy evaluation experiments (*n* = 42). Tumors excised from mice engrafted patient tumors were defined as the first generation (G1), and the generations of tumors excised at passaging were defined as G2, G3, and G4, in that order. These characteristics were evaluated using morphology, immunohistochemistry (IHC), and droplet digital PCR. CTOS was established according to the method previously described [13, 14], using a portion of the G3 tumor of PDX in this study. Briefly, tumors were minced and digested using Liberase™ DH (Roche, Basel, Switzerland), after which CTOS size fractions of 100- or 40- μm were collected using a cell strainer. Fractions were cultured in StemPro™ hESC SFM (Life Technologies, Carlsbad, CA) supplemented with 8 ng/mL bFGF (Life Technologies, Carlsbad, CA) and 0.1 mM 2-mercaptoethanol (Wako Chemical Co., Tokyo, Japan). CTOS were established as CTOS lines after at least two in vivo passages of subcutaneous transplantation of CTOS into BRJ mice under anesthesia, and CTOS in the 40–70 μm size fraction were used for evaluation of drug efficacies.

### Immunohistochemistry

Patient tumor samples and tumors excised from patient-derived models were stained with IHC and Hematoxylin Eosin (HE). Primary antibodies used for IHC were as follows: HER2 (HER2/neu, Agilent Technologies, Glostrup, Denmark), CK7 (OV-TL12/30, Agilent Technologies, Glostrup, Denmark), CK20 (Ks20.8, Agilent Technologies, Glostrup, Denmark), and GATA3 (L50–823, Cell Marque, The Hague, Netherlands). Glass slide samples were digitized with a NanoZoomer S60 digital slide scanner (Hamamatsu Photonics, Hamamatsu, Japan).

### Droplet digital PCR for analyzing *HER2* copy number and the ratio of mutant and wild type *HER2*

Droplet digital PCR (ddPCR™) for analyzing the ratio of E401G and wild type *HER2* was performed as described previously [10]. Copy number assay using ddPCR was performed with a *HER2* probe set (dHsaCP1000116, Bio-Rad Laboratories, Inc., USA) and the reference gene, RPP30, probe set (dHsaCP2500350, Bio-Rad Laboratories, Inc., USA), according to the manufacturer’s protocol.

### Animal studies

To explore appropriate treatment for the patient, we examined drug efficacy using PDX described above (*n* = 6 per group). We also used xenograft using H2170 cells with wild-type *HER2* amplification to compare the effects with PDX with *HER2* E401G amplification (*n* = 3 or 4 per group). H2170 cells (1 × 10^7^ each) were injected into the dorsal flanks of 7-week-old female BRJ mice under anesthesia with midazolam, medetomidine, and butorphanol tartrate. Tumor sizes were measured with calipers twice per week and tumor volumes were calculated as *V* = 1/2 × [(the shortest diameter)^2^ × (the longest diameter)]. For drug efficacy evaluation, drug or vehicle administration was started on the day that the tumor size reached 150–200 mm^3^ for PDX and 100–200 mm^3^ for H2170 xenograft (in both cases defined as “day 1”). On day 22, mice were euthanized by cervical dislocation and excised tumors were photographed. Regarding the administration of the drugs, sub-maximal tolerated doses of afatinib and lapatinib were reported to be 20–25 mg/kg [11, 12, 15] and 100–150 mg/kg [11, 16, 17], respectively, so the initial experiments were performed with 20 mg/kg of afatinib and 100 mg/kg of lapatinib. However, due to a lack of efficacy in the lapatinib 100 mg/kg group, a lapatinib 150 mg/kg group was added. Afatinib and lapatinib were prepared in 0.5 w/v (%) methyl cellulose and administered orally once daily for 5 days per week. Trastuzumab and pertuzumab were administered intraperitoneally once a week at 30 mg/kg diluted in saline according to the literature published during the time of their development [18, 19]. PDX excised tumors were cryopreserved and analyzed for *HER2* copy number by droplet digital PCR. In each experiment, the mice were randomly assigned into two or three groups. Mice were maintained in a specific pathogen-free facility within the Analytical Research Center for Experimental Sciences, Saga University. Mice were euthanized by cervical dislocation when a loss of more than 20% baseline body weight occurred or other human endpoints such as lethargy. All animal studies were performed in accordance with animal research protocols approved by the Institutional Review Board of Saga University (A2021–022-0) and the university’s institutional guidelines. The study was completed and reported in compliance with the recommendations of Animals in Research: Reporting In Vivo Experiments (ARRIVE) guidelines.

### Drug sensitivity assay using CTOS

CTOSs prepared by the method described above were counted and seeded into 96-well plates at approximately 10 CTOSs/well. Twenty-four hours later, medium containing each drug was added to each well to achieve the targeted final concentration, then the culture was continued. Seven days after the addition of the drugs, ATP content was measured with CellTiter-Glo Luminescent Cell Viability Assays (Promega, Madison, WI, USA), and the content was adjusted with vehicle-treated control.

### Drug sensitivity assay in vitro

H2170 cells were seeded into 96-well plates at 7 × 10^3^ cells/well; 24 hours later each drug was added, then the culture was continued. Cell growth was measured with a Cell Counting Kit-8 (Dojindo Molecular Technology, Kumamoto, Japan) on the third day after the addition of the drugs, and the content was adjusted using vehicle-treated control.

### Statistical analysis

Data are expressed as mean and standard deviation (SD). Differences between two groups were tested with the Wilcoxon rank-sum test. Differences among three groups were tested with the Kruskal-Wallis test and Dunn’s test for pairwise comparisons. For all comparisons, *P* < 0.05 was considered statistically significant. Most calculations were performed using JMP Pro 15.2.0 (SAS Institute Inc., USA). Dose response curves were plotted and half maximal inhibitory concentration (IC_50_) values were calculated with the DRC package (version 3.0.1) for R (version 4.1.3) in RStudio (version 2022.2.1.461; RStudio PBC, Boston, Massachusetts, USA).

## Results

### Clinical course of a patient with coexisting *HER2* E401G mutation and *HER2* amplification

**Fig. 1:**
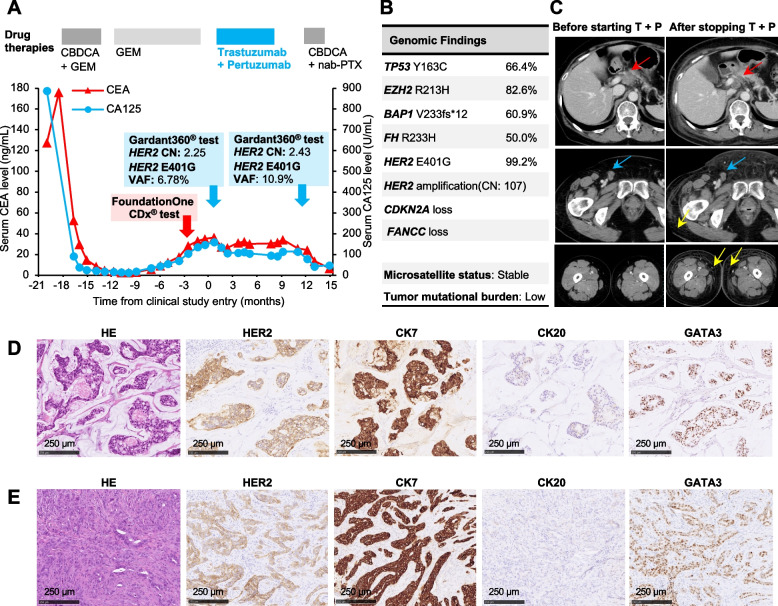
Clinical course and tumor profile of a patient with cancer bearing an amplified *HER2* E401G mutation. **A** Clinical course before and after basket trial with trastuzumab and pertuzumab. Carcinoembryonic antigen (CEA) and cancer antigen 125 (CA125) are tumor markers that reflect the disease status of the patient. Bold arrows indicate the timing of the multi-gene panel tests with FoundationOne CDx® (red arrow) and Gardant360® (blue arrows). CBDCA, carboplatin; GEM, gemcitabine; nab-PTX, nab-paclitaxel; VAF, variant allele fraction. **B** Results of FoundationOne CDx® test, a multi-gene panel test. Among the detected variants, only those considered pathogenic are shown. Gene amplifications interpreted as equivocal (four copies) are not shown. **C** Results of computed tomography. The left image is before start of trastuzumab and pertuzumab combination therapy (T + P); the right image is after discontinuation of therapy. Red arrows indicate abdominal lymph node metastases; blue arrows indicate inguinal lymph node metastases. Yellow arrows point to skin edema thought to be associated with skin invasion by cancer. **D** Pathological findings of cancer tissue (inguinal lymph node biopsy specimen) at the time of diagnosis of cancer. **E** Pathology findings of skin biopsy specimen when skin invasion of cancer was confirmed, before T + P was discontinued

The clinical course of a 67-year-old Japanese woman with carcinoma of unknown primary up to enrollment in the JUPITER trial (jRCT2031180150) [20], a basket trial of trastuzumab and pertuzumab combination therapy, was described in detail in our previous report [10]. After initial treatment with carboplatin and gemcitabine, the patient’s cancer gradually became refractory to the therapy. The FoundationOne CDx® test, a multi-gene panel test, showed *HER2* amplification with coexisting extracellular domain *HER2* E401G variant (Fig. 1A, B). Because the patient met the JUPITER trial eligibility criteria of *HER2* amplification detected by an NGS-based assay, she was enrolled into the trial and received the combination of trastuzumab and pertuzumab. The best overall response to treatment according to RESIST version 1.1 [21] was “Stable Disease” with a certain degree of tumor control but not sufficient tumor volume reduction (Fig. 1C). Eight months later, skin metastasis was observed and diagnosed by skin biopsy, which pathological findings similar to those at initial diagnosis (Fig. 1D, E); treatment in the trial was therefore discontinued.

As an ancillary part of the trial, liquid biopsy using Gardant360**®**, a multi-gene panel test, was performed at the time of enrollment and at the end of treatment in clinical trials. In this test, a copy number (CN) of 2.2 or higher is considered to indicate a clinically significant increase in the CN of the gene [22]. The results in this patient showed a *HER2* CN of 2.25 and *HER2* E401G variant allele fraction (VAF) of 6.78% at the time of enrollment. At the end of treatment in the clinical trial, CN was 2.43 and E401G VAF was 10.9%, with a slightly increasing trend reflecting disease progression (Fig. 1A). Based on the Ethics Committee review and the patient’s written informed consent, we initiated investigation to determine the next treatment regimen, by using PDX and other approaches, utilizing a portion of the skin biopsy tissue.

### PDX and CTOS models retained characteristics of original patient tumor

**Fig. 2:**
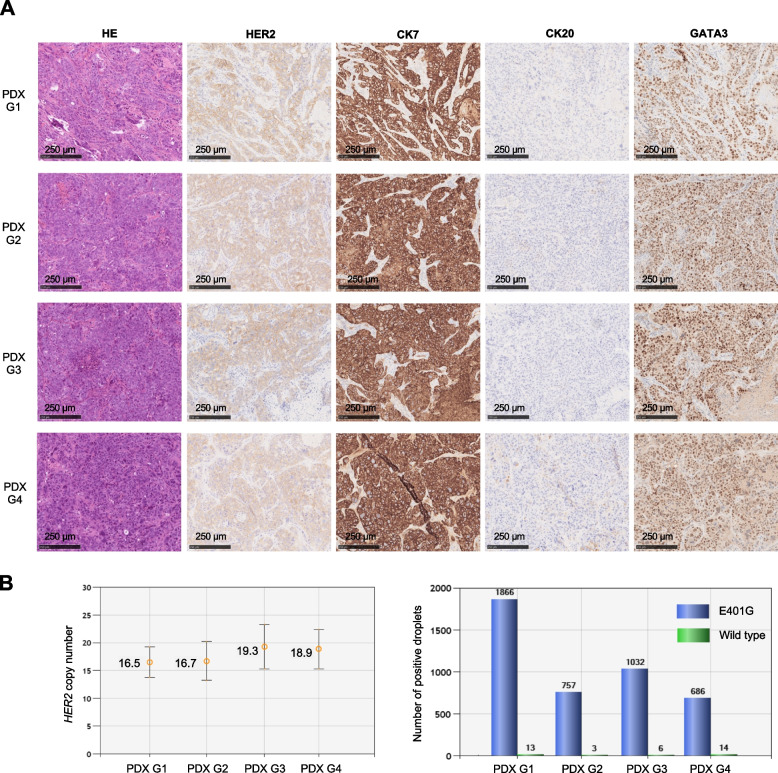
Pathological and genetic profiles, during in vivo passages, of xenograft derived from the patient’s cancer. **A** Morphological and immunohistochemical staining results during passaging of patient derived xenografts (PDX). Tumors excised from mice with engrafted patient tumors were defined as the first generation (G1), and the generations of tumors excised at passaging were defined in order as G2, G3, and G4. HE, Hematoxylin Eosin stain; HER2, human epidermal growth factor receptor 2; CK7, Cytokeratin 7; CK20, Cytokeratin 20; GATA3, GATA binding protein 3. **B** *HER2* copy number and ratio of *HER2* E401G to wild type by droplet digital PCR. The left figure shows the result of *HER2* copy number assay; the right figure shows the result of mutation detection assay to check the mutant-to-wild-type ratio

**Fig. 3:**
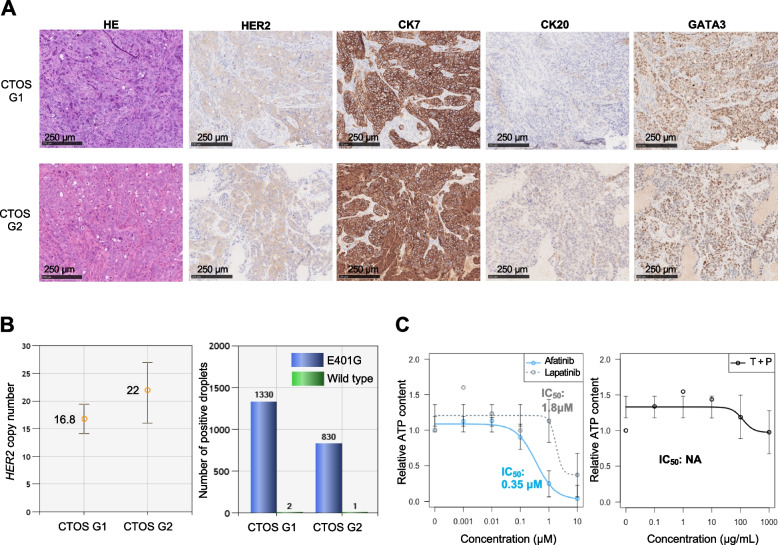
Pathological and genetic profiles during the process of cancer-tissue originated spheroid (CTOS) preparation and use of CTOS to evaluate drug efficacy. **A** Morphological and immunohistochemical staining results during preparation of CTOS. CTOS were established as CTOS lines after at least two in vivo passages of subcutaneous transplantation of CTOS into BRJ mice. The first generation of succeeding tumors in in vivo passage was denoted as CTOS G1 and the second generation as CTOS G2. HE, Hematoxylin Eosin stain; HER2, human epidermal growth factor receptor 2; CK7, Cytokeratin 7; CK20, Cytokeratin 20; GATA3, GATA binding protein 3. **B** *HER2* copy number and *HER2* E401G-to-wild-type ratio by droplet digital PCR. The left figure shows the result of *HER2* copy number assay; the right figure shows the result of mutation detection assay to check the mutant-to-wild–type ratio. **C** Drug efficacy evaluation using CTOS. IC_50_, half maximal inhibitory concentration; T + P, trastuzumab and pertuzumab

We used histological and genetic approaches to evaluate whether the PDX models retained the characteristics of the original tumor. Evaluation of drug efficacy in PDX was performed after at least three in vivo passages by transplantation into BRJ mice, and the morphology and IHC profiles of the tumors excised during passages (PDX G1, G2, G3 and G4) were similar to those of the original tumor (Fig. 2A). In addition, the *HER2* CN did not change during in vivo passages, and E401G remained more frequent than wild type in the amplified *HER2* gene (Fig. 2B). In addition, CTOS model preparation was performed concurrently with PDX establishment for evaluation in a distinct experimental system. After the initial CTOS preparation process, we used CTOS obtained after two in vivo passages in BRJ mice for drug efficacy evaluation. As was the case with PDX, tumors (CTOS G1 and G2) during passages in the CTOS preparation process retained the histological and genetic characteristics of the tumors (Fig. 3A, B).

### Afatinib was the most effective drug in the CTOS model

Initially, we evaluated the efficacy of the drugs in the CTOS model for cancers with the *HER2* E401G and its amplification. Afatinib, a TKI with an inhibitory effect on EGFR in addition to well as on HER2, was the most effective in suppressing tumor growth, with an IC_50_ of 0.35 μM (Fig. 3C). On the other hand, lapatinib, an anti-HER2 TKI with a weaker inhibitory effect on EGFR than afatinib, was less effective in inhibiting tumor growth (IC_50_: 1.8 μM) (Fig. 3C). The effect of trastuzumab plus pertuzumab (T + P), for which there is clinical evidence of efficacy against cancers with wild-type *HER2* amplification, was very weak (Fig. 3C). Because both trastuzumab and pertuzumab are anti-HER2 monoclonal antibody drugs, antibody-dependent cellular cytotoxicity (ADCC) and complement-dependent cytotoxicity in vivo are also involved in their efficacy. The effect of T + P was considered necessary to validate these drugs in the in vivo model.

### Afatinib was also the most effective drug in the PDX model

**Fig. 4:**
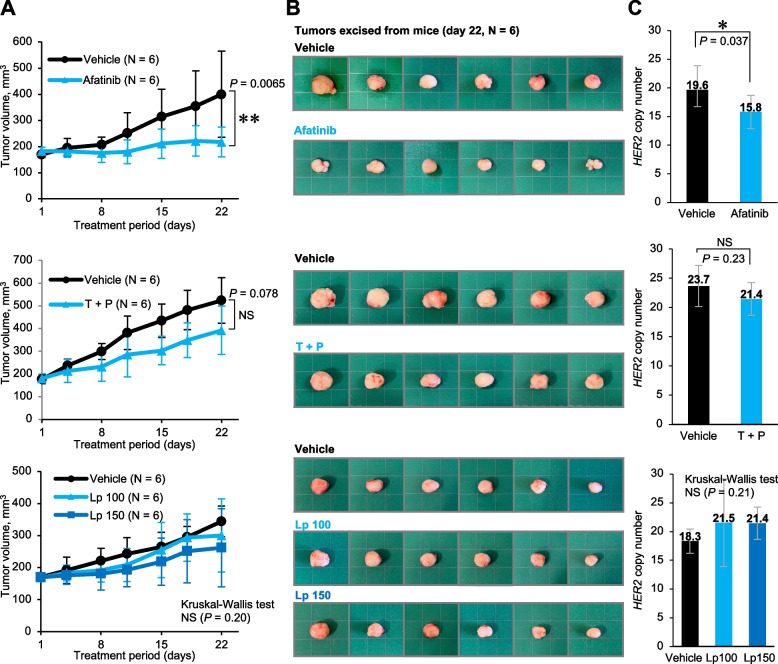
Evaluation of drug efficacy using xenografts derived from patient’s cancer bearing amplified *HER2* E401G. **A** Tumor size in each drug group and vehicle group in patient-derived xenografts (PDX). Error bars represent SD. *P < 0.05 and **P < 0.01. T + P, trastuzumab and pertuzumab; Lp 100, Lapatinib 100 mg/kg; Lp 150, Lapatinib 150 mg/kg. **B** Appearance of tumors at the time of sacrificed (day22). **C** *HER2* copy number at the end of treatment. DNA was extracted from the tumor excised on day 22 and *HER2* copy number was analyzed by droplet digital PCR

**Fig. 5:**
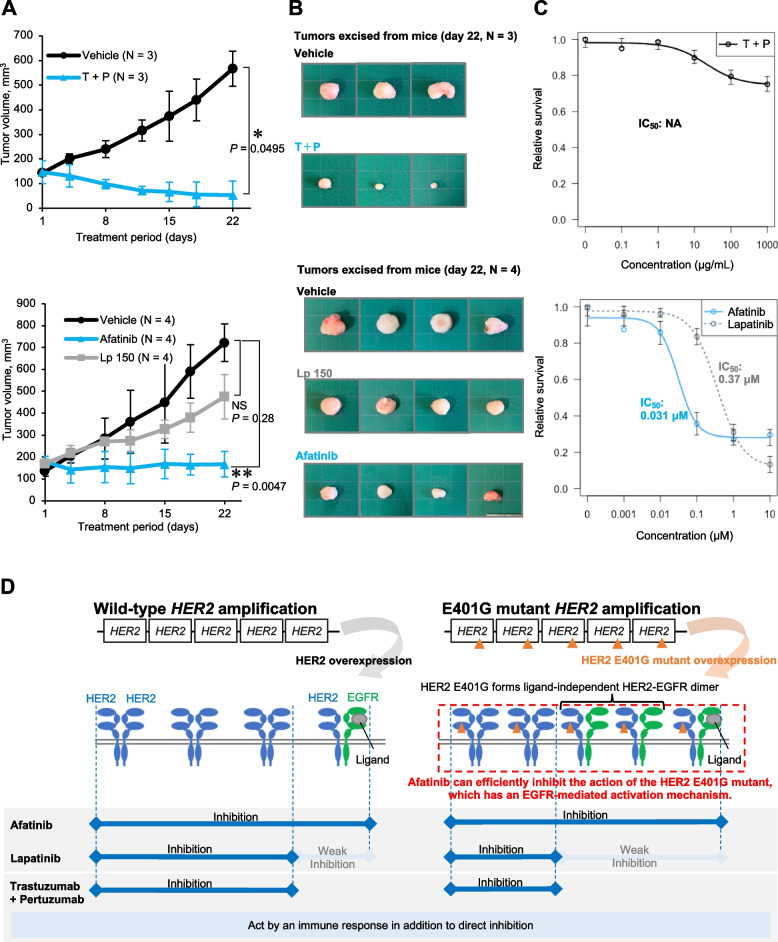
Evaluation of drug efficacy using xenografts of H2170 cells with amplification of wild-type *HER2* and summary of our arguments by schema. **A** Comparison of tumor size in each drug group and vehicle group in H2170 xenografts. Differences between the two groups were tested with the Wilcoxon rank-sum test. Differences among three groups were tested with the Kruskal-Wallis test and Dunn’s test. Error bars, SD. *P < 0.05 and **P < 0.01. T + P, trastuzumab and pertuzumab; Lp 150, Lapatinib 150 mg/kg. **B** Appearance of tumors at the time when the mice were sacrificed (day22). **C** Drug efficacy evaluation using H2170 cells in vitro. IC_50_, half maximal inhibitory concentration. **D** Summary of our arguments by schema. HER2, human epidermal growth factor receptor 2; EGFR, epidermal growth factor receptor; TKI, tyrosine kinase inhibitor

Next, we evaluated the efficacy of the same drugs in the in vivo PDX models. Afatinib was the most effective among all treatments, with a statistically significant inhibition of tumor growth on day 22 after initiation of drug treatment (Fig. 4A, B). T + P tended to suppress tumor growth, but no statistically significant difference was observed. Lapatinib showed less inhibition of tumor growth in both the 100 mg/kg and 150 mg/kg dose settings. In addition, *HER2* copy number in tumors in each mouse on day 22 after anti-HER2 therapy decreased was significantly lower in the afatinib-treated group than in the vehicle-treated group, but was not significantly lower with the other treatments (Fig. 4C). To compare the difference in efficacy with and without E401G in coexistence with *HER2* amplification, we evaluated the drug efficacy on H2170 cells, a lung cancer cell line with wild-type *HER2* amplification. The results showed that T + P was profoundly effective against H2170 cells in vivo and provided the greatest effect of all drugs (Fig. 5A, B). This finding was contrary to the results in the cancer model with coexisting E401G mutation. Afatinib also produced statistically significant tumor shrinkage in H2170, and its effect was superior to that of lapatinib (Fig. 5A, B). The trend was similar in vitro, although efficacy of T + P were relatively weak (Fig. 5C).

## Discussion

In this study, we demonstrated that afatinib, a tyrosine kinase inhibitor that is effective against both HER2 and EGFR, showed an anti-cancer effect for *HER2* E401G and its amplification using the cancer models PDX and CTOS derived from a patient tissue specimen. These results support the hypothesis that suppression of EGFR signaling simultaneously with HER2 could be effective against cancers with the *HER2* E401G mutation, based on our previous results of in vitro analyses and in silico molecular dynamics simulation in which HER2 activation was mediated through stabilization between EGFR and HER2.

As is often the case, results with cell lines used for evaluation of drug efficacy were not identical to clinical outcomes [23, 24]. This is likely due to changes in genetic profiles and gene expressions during the process of cell line establishment [25, 26]. Therefore, PDX and other patient-derived cancer models have been studied and there are many reports suggesting that these models reflect therapeutic efficacy in clinical practice [27–29]. However, PDX requires a relatively long time to establish and has a low throughput, allowing study of the effects of only a limited number of drugs. Recently, organoid culture methods have been developed as a new model to fill this gap [30, 31]. One of them is CTOS, a high-yield method that produces highly pure organoids [13, 32, 33]. Therefore, we used PDX and CTOS to evaluate drug efficacies for cancer with amplified *HER2* E401G. We confirmed that morphological and immunohistochemical characteristics of the cancer, as well as *HER2* gene profiles in the original tumors, were maintained in PDX and CTOS-established tumors.

In the evaluation of drug efficacy in the present study, different results were demonstrated between the cell line, H2170, and the patient-derived cancer model. As a premise of our conclusion, the difference in drug efficacy due to the presence of *HER2* E401G is presumably due to factors other than a mutation-induced change in drug binding. Because afatinib and lapatinib bind to the target kinase domain, whereas pertuzumab and trastuzumab bind to HER2 extracellular domains 2 and 4, respectively [34], the *HER2* E401G mutation site (extracellular domain 3) and the binding sites of these drugs are different. T + P produced remarkable tumor shrinkage of xenografts in the H2170 cell line with wild-type *HER2* amplification, whereas the PDX model with E401G co-existing with *HER2* amplification produced only a small effect. The best therapeutic effect of T + P on the patient from whom cancer tissue was obtained was “stable disease”, and tumor shrinkage was not observed, as shown in Fig. 1. We previously showed that the biological effect of E401G on HER2 activation was similar to that of S310F, a hot spot of *HER2* extracellular domain mutation. A report on the evaluation of drug efficacy in a PDX model established from patients with cancers harboring coexisting *HER2* S310F mutation and *HER2* amplification showed that afatinib was markedly superior to trastuzumab plus lapatinib and other agents [35]. This suggests that the coexistence of *HER2* E401G mutation with *HER2* amplification may attenuate the therapeutic effect of targeting HER2 alone.

We also showed that afatinib was superior to lapatinib and T + P in both PDX and CTOS models with amplified *HER2* E401G mutation. In addition, there was a statistically significant reduction in *HER2* copy number in PDX tumor only with afatinib treatment. Both lapatinib and afatinib are selective ATP-competitive inhibitors of EGFR and HER2 [36]. The IC_50_ for EGFR and HER2 in a cell-free in vitro kinase assay were 3 nM and 15 nM for lapatinib and 0.5 nM and 14 nM for afatinib, respectively, showing that afatinib is more potent in its inhibitory activity on EGFR [12]. In addition, lapatinib has a reversible binding mode to the receptor [36], whereas afatinib has an irreversible binding mode to the receptor through covalent binding, resulting in a long-lasting inhibitory effect [37]. Based on the different properties of these agents, it is possible that afatinib, due to its potent and prolonged inhibition of EGFR in addition to its inhibition of HER2, provided a better effect than lapatinib on cancer with *HER2* E401G. The decrease in *HER2* copy-number is presumably due to a decrease of the average copy number in cancer cells as a result of HER2-targeted therapy. High efficacy of anti-HER2 therapy reduced the number of clones with high *HER2* copy number, resulting in reduction of the average copy number in all cancer cells. The effects of anti-HER2 therapies on *HER2* wild type and E401G mutants, with amplification, are summarized in Fig. 5D. Possible mechanisms for the effects are also illustrated.

We acknowledge several limitations in our study. First, we did not perform experiments to evaluate whether similar results are obtained with other extracellular domain mutations such as S310F. However, other groups have already reported significant efficacy of afatinib in PDX with *HER2* S310F and in patients [35]. Second, we could not provide molecular evidence that EGFR inhibition is a key factor in the efficacy of afatinib against cancers with *HER2* E401G mutation, due to the limited tissue and necrosis. Third, we have not conducted any additional combination experiments of afatinib with T + P. Theoretically, these combinations could produce additional benefits, but afatinib is a drug that requires management of side effects, such as skin toxicity and diarrhea, so adding drugs might not be feasible. In fact, in a phase I study of the combination of afatinib and trastuzumab, side effects such as diarrhea were a major problem [38]. Our study not only demonstrates that afatinib is a promising therapeutic option for the present variant combination, but also highlights the importance of determining the activation mechanism of the mutations and developing therapies based on such findings.

## Conclusions

Our study provides evidence that treatment strategies that suppress EGFR signaling simultaneously with HER2 are effective for *HER2* E401G mutations with an EGFR-mediated activation mechanism. Our study provides important clues for implementing patient-specific precision medicine in clinical oncology practice.

## Abbreviations

NGS: Next-generation sequencing
HER2: human epidermal growth factor receptor 2
VUS: variants of unknown significance
EGFR: epidermal growth factor receptor
TKI: tyrosine kinase inhibitor PDX patient-derived xenograft
CTOS: cancer tissue-originated spheroid
BRJ: Balb/c Rag-2^−/−^ Jak3^−/−^
G: generation
IHC: immunohistochemistry
SD: standard deviation
IC_50_: half maximal inhibitory concentration
CN: copy number
VAF: variant allele fraction
T + P: trastuzumab plus pertuzumab
ADCC: antibody-dependent cellular cytotoxicity.
